# Chromosome-contiguous nuclear genome of *Dirofilaria asiatica* reveals unique molecular signatures and host interactions

**DOI:** 10.1016/j.onehlt.2025.101201

**Published:** 2025-09-12

**Authors:** Neil D. Young, Yuanting Zheng, Anson V. Koehler, Tao Wang, Sunita B. Sumanam, Ushani Atapattu, Bill C.H. Chang, Vito Colella, Robin B. Gasser

**Affiliations:** Department of Veterinary Biosciences, Melbourne Veterinary School, The University of Melbourne, Parkville, VIC 3010, Australia

**Keywords:** *Dirofilaria asiatica*, Chromosome-scale nuclear genome, Filarioid nematode, Immune evasion, Excretory-secretory proteins, Host–parasite interactions, Zoonosis, Molecular epidemiology, Long-read sequencing, High-throughput chromosome conformation capture (Hi-C), Comparative genomics, One Health

## Abstract

*Dirofilaria asiatica* is a recently described filarioid nematode of zoonotic importance whose biology, host interactions and epidemiology are largely unknown. Here, we present the first chromosome-scale nuclear genome for this species, assembled from long-read PacBio and short-read Hi-C (high-throughput chromosome conformation capture) sequence data derived from adult specimens. The resulting 91.9 Mb genome comprises four autosomes and one sex-linked scaffold and encodes 9658 protein-coding genes with a high level of completeness. Comparative genomic analyses with *Dirofilaria immitis*, *Brugia malayi* and *Onchocerca volvulus* revealed both conserved chromosomal synteny and lineage-specific rearrangements. We identified 881 predicted excretory/secretory (ES) proteins, with a marked enrichment in immune-relevant pathways, including proteolysis, lysosomal activity and antigen presentation. Notably, 229 (∼26 %) of these ES proteins were unique to *D. asiatica*, many of which are implicated in host–parasite interactions, immune evasion and metabolic adaptation in the mammalian host. Proteins such as cystatins, serpins, venom allergen-like (VAL) proteins and cytokine-mimics suggest specialised immunomodulatory capacities distinct from those of other filarioids. The genome of *D. asiatica* fills a significant gap in filarial genomics and provides a foundational resource for future studies into host adaptation, molecular epidemiology, and the development of improved diagnostics and interventions, with direct relevance to a One Health approach for monitoring and controlling filarioid infections at the human–animal–vector interface.

## 1 Introduction

Nematodes are among the most speciose phyla of multicellular organisms (Metazoa) on Earth [1]. More than 27,000 species have been formally described, but hundreds of thousands are likely to exist [2,3]. The majority (∼80 %) are free-living and play vital ecological roles in soil, freshwater and marine environments, while the remainder (∼20 %) are parasitic in plants or animals, including humans [3]. Among the parasitic nematodes, filarioid species (family Onchocercidae) constitute a particularly important group of vector-borne pathogens, many of which have major adverse impacts on public and veterinary health [4]. These nematodes are transmitted by blood-feeding arthropods and often exhibit complex life cycles and tissue tropism, including lymphatic, subcutaneous, ocular and cardiopulmonary localisations.

In humans, *Wuchereria bancrofti* and *Brugia* spp. cause lymphatic filariasis (including elephantiasis), affecting an estimated 50 million people worldwide, while *Onchocerca volvulus* is the causative agent of onchocerciasis (river blindness), with more than 20 million people infected, predominantly in sub-Saharan Africa [5]. Several related filarioids of the genus *Dirofilaria* primarily infect domestic and wild carnivores, but some are zoonotic and capable of causing disease in humans. Of the more than 27 species of *Dirofilaria* currently recognised, at least six have been implicated in zoonotic infections [6,7]. *Dirofilaria repens*, which causes subcutaneous and ocular lesions in canids, is the predominant agent of human dirofilariasis in Europe, Asia and Africa (“Old World”), whereas *D. immitis*, the causative agent of heartworm disease in dogs, can cause zoonotic pulmonary dirofilariasis in the Americas (“New World”). However, cases of imported infections due to increased international travel and the translocation of pets and people are now being reported across a widening geographic range [7], illustrating the interconnectedness of animal, human and vector health and the growing need for an integrated One Health focus.

Despite their importance, many aspects of *Dirofilaria* biology and epidemiology remain poorly understood. The lack of reliable morphological characters for differentiating closely related species, coupled with limited availability of molecular data for most taxa, continues to hamper accurate species identification and comparative studies. In 2012, a distinct *Dirofilaria* genotype – detected in nodular lesions in human patients and in blood of samples from stray dogs in Hong Kong – was proposed as a novel species [8,9]. However, owing to the absence of a formal morphological diagnosis, the name was a nomen nudum under the International Code of Zoological Nomenclature (ICZN) [9]. This parasite was subsequently reported in human patients from India and Thailand, in travellers returning to Germany and Austria from India, and in patients in Australia who had migrated from Sri Lanka [[10], [11], [12], [13], [14]]. It was inferred to cause subcutaneous or subconjunctival dirofilariasis in humans, with dogs suspected to serve as a reservoir host [15]. Nevertheless, the biological significance and zoonotic potential of this genotype or taxon remained poorly defined.

Recently, we formally described this lineage as *Dirofilaria asiatica*, using an integrative approach that combined detailed morphological characterisation with mitochondrial genome sequencing of adult worms derived from naturally infected dogs in Puttalam, Sri Lanka [16]. While mitochondrial data provided preliminary insight into its phylogenetic placement within *Dirofilaria*, mitochondrial genomes alone are insufficient to characterise its molecular biology and host–parasite interactions. A high-quality nuclear genome is needed to explore these aspects in detail and to place *D. asiatica* within the broader evolutionary and biological context of filarioid nematodes.

Advances in long-read and in situ chromatin conformation capture (Hi-C) sequencing technologies [17] are transforming molecular research of parasitic nematodes. For several filarioids – including *D. immitis, Brugia malayi* and *O. volvulus*, high-quality, chromosome-scale genome assemblies [[18], [19], [20], [21], [22], [23], [24]] have paved the way for detailed analyses of gene content, structural variation and evolution. However, many newly recognised, geographically restricted or neglected species remain underrepresented in genomic databases. This paucity of nuclear genome data restricts comparative genomic analyses and limits efforts to identify molecular markers for improved diagnosis and intervention.

*Dirofilaria asiatica* exemplifies this gap. To address this issue, we generated the first chromosome-level nuclear genome for *D. asiatica*. Genomic DNA was extracted from single worm specimens derived from tissue nodules, demonstrating the feasibility of using archived parasitological material for high-resolution sequencing. We applied Pacific Biosciences (PacBio) long-read sequencing in combination with chromosome conformation capture (Hi-C) to assemble a contiguous and structurally resolved genome. This resource provides a foundation for downstream gene annotation, comparative analyses and investigations into molecular pathways, including those underpinning host–parasite crosstalk. The successful assembly and annotation of a high-quality nuclear genome from minimal input material emphasises the value of curated specimen archives and the power of third-generation sequencing technologies. This nuclear genome now offers a platform for future studies into the developmental biology and reproduction, vector and host associations and epidemiology of *D. asiatica* – all of which are presently unknown but are central to assessing zoonotic potential and informing a One Health framework for integrated control.

## 2 Materials and methods

### 2.1 Adult worms of *Dirofilaria asiatica*

Animal ethics approval (permit: VERC/20/07) was granted by the Committee for Ethical Clearance on Animal Research of the Faculty of Veterinary Medicine and Animal Science, University of Peradeniya, Sri Lanka. Adult *D. asiatica* were collected from nodules within the testes, scrotum and/or spermatic cords of dogs in Puttalam, Sri Lanka. Nodules were excised and stored in 70 % (*v*/v) ethanol or RNAlater (Thermofisher, USA) at −20 °C. Subsequently, specimens were examined by light microscopy [16] prior to nucleic acid isolation.

### 2.2 Isolation of genomic DNA and sequencing

Following extensive washing in sterile physiological saline, high quality genomic DNA was isolated from the mid-body sections of intact individual male and female worms from the ethanol-preserved nodules using an established protocol [25]. An aliquot of genomic DNA from each individual worm used was subjected to polymerase chain reaction (PCR)-based sequencing of a portion of the cytochrome *c* oxidase subunit 1 (cox-1) gene [26] to confirm its identity as *D. asiatica*, and to demonstrate that it was genetically consistent with the “Hong Kong” genotype of *Dirofilaria* [8].

First, PacBio sequencing was conducted. Genomic DNA samples from a single male worm (6 ng) and a single female worm (216 ng) were each subjected to whole genome amplification using the REPLI-g midi kit (Qiagen, Germany); the quantity and quality of this amplified DNA was assessed in the TapeStation system (Agilent 4200) using Genomic DNA ScreenTape (Agilent). An aliquot (25 μg) of each of these two DNA samples was used to construct a SMRT library (PacBio Revio). The two libraries were then sequenced using the PacBio Revio Platform at BGI Limited in Hong Kong, with sequence data stored in the FASTQ format.

Second, Hi-C sequencing was performed. Two genomic DNA samples prepared from pools of six RNAlater-preserved male worms (wet weight: 50 mg) and six female worms (100 mg) were used to construct libraries employing the Arima High Coverage Hi-C kit (Arima Genomics, CA, USA; L/N2309050008; part no. A160162 v01). Both libraries were assessed for quality using the TapeStation and paired-end sequenced in one lane of a NovaSeq X (10B, 300 cycles; Illumina, USA), with short reads stored in the FASTQ format.

### 2.3 Genome assembly

To obtain a draft genome for *D. asiatica*, PacBio long reads derived from both SMRT libraries were assembled using hifiasm v.0.19.8 [27]. Then, haplotypic sequences were removed from the assembly using purge_haplotigs v.1.1.0 [28]. Chromosome-length scaffolds were obtained from the Hi-C data set using Chromap v.0.2.5 [29], YaHS v.1.1 [30], and HiContacts v.1.0 [31] and Juicebox v.2.20.00 [32] were used for scaffold analysis and visualisation. Gaps in scaffolds were closed using error-corrected long reads employing the program DENTIST v.4.0.0 [33]. At each step, assembly results were assessed using QUAST v.5.2.0 [34] and BUSCO v.5.1.2 [35], and the parameters of the various software tools were optimised to achieve a chromosome-contiguous assembly.

### 2.4 Isolation of RNA and sequencing

Total RNA was isolated separately from two male worms (152 ng) and two female worms (540 ng) stored in RNA later using the Tripure isolation reagent (Roche). The RNA quantity of each of the two samples was assessed using the Qubit RNA High Sensitivity Assay Kit (Life Technologies, Carlsbad, CA, USA), and their quality and integrity evaluated using the TapeStation employing RNA Screen Tapes (Agilent, CA). Two aliquots (60 ng for male, and 180 ng for female) were used to construct two separate cDNA libraries using the SQK-PCB114.24 kit, which were sequenced using nanopore technology in a flow cell (FLO-PRO114M) in the Promethion 2 Solo platform (Oxford Nanopore Technologies, Oxford, UK). Data were stored in the POD5 file format for subsequent “base-calling” using the program Dorado release 0.8.3 (Oxford Nanopore Technologies, Oxford, UK), and sequence reads were stored in the FASTQ format.

### 2.5 Prediction and functional annotation of protein-coding genes

First, custom repeat models, inferred from the *D. asiatica* draft genome using RepeatModeler v.2.0.4 [36] and Terrier v.0.2.0 [37], were masked in the assembled genome utilising RepeatMasker v.4.1.5 [38]. Then, gene models were predicted from the masked genome using a combination of bioinformatic tools or pipelines, including Braker v.3.0.3 [39,40], Funannotate v.1.8.1 [41] and PASA v.2.5.3 [42], supported by RNA and proteomic evidence from *B. malayi* (NCBI accession: GCA_000002995.5), *O. volvulus* (accession: GCA_000499405.2) and *D. immitis* (accession: GCA_024305405.1). The designation and orientation of individual chromosomes corresponded to those of *B. malayi* inferred using GENESPACE v.1.2.3 [43]. The completeness of the gene set was assessed using BUSCO v.5.1.2 for 3131 genes [35] and OMArk v.0.3.0 [44]. Genes/inferred proteins were annotated using established pipelines [45,46], which incorporates InterProScan v.5.6.1 [47] and Eggnog-mapper v.2.1.9 [48], and presented in the GFF3 format and then refined to comply with NCBI submission requirements employing programs AGAT v.1.2.0 [49] and gffread v.0.12.7 [50].

### 2.6 Synteny and genome comparisons

Using OrthoFinder v.2.5.4 [51], single-copy and one-to-one (1:1) orthologues were identified. These genes were employed to investigate genuine synteny and orthology between *D. asiatica* and the genomes of related onchocercids for which high quality genomes are available (i.e. *D. immitis, B. malayi* and *O. volvulus*). Circos plots were produced using the program shinyCircos-v.2.0 [52] using a sliding window of 100 kb.

### 2.7 Identification, annotation and comparative analyses of selected gene and protein sets

Excretory–secretory (ES) proteins of *D. asiatica* were inferred using an established bioinformatic pipeline [45], incorporating Phobius v.1.0.0 [53,54] to identify proteins containing a signal peptide but lacking transmembrane domains. Proteins were functionally annotated using eggNOG-mapper v.2.1.9 [48] and InterProScan v.5.57–90 [47], which also provided complementary information including Gene Ontology (GO) terms [55], Enzyme Commission (EC) numbers [56], Pfam domains [57] and Kyoto Encyclopedia of Genes and Genomes (KEGG) pathway associations [58]. GO annotations were retrieved from the GO-basic.obo file (release 2022-09-19), and pathway mapping was conducted using TBtools v.1.0987663 [59]. The same workflow was applied to predict ES proteins from the genomes of *D. immitis, B. malayi* and *O. volvulus*. OrthoFinder v.2.5.4 [51] was employed to define orthologous groups (orthogroups) of proteins within and among species. In addition, we used the Hidden Markov Model (HMM) to scan (in 10,000 non-overlapping windows) the *D. asiatica* genome (translated in six frames) for genes encoding CAP domain-containing proteins (PF00188) employing hmmsearch v.3.2.2 [60]. The number and identity of these genes were compared with those reported for *D. immitis, B. malayi* and *O. volvulus* [61], and homologues were identified in *C. elegans* using blastp v2.5.0 [62].

### 2.8 Analysis of transcription

For each of the RNA samples representing individual adult specimens of *D. asiatica* (designated female 1 [F1; SQK-PCB114-24_barcode11]; female 2 [F2; SQK-PCB114-24_barcode12]; male 1 [M1; SQK-PCB114-24_barcode13] and male 2 [M2; SQK-PCB114-24_barcode14]), long-read sequences were filtered according to length and quality and then mapped to the reference genome of *D. asiatica* using minimap2 v.2.26 [63]. Transcripts were counted using StringTie2 v.2.2.3 [64], and the fragments per kilobase of transcript per million mapped reads (FPKM) values were calculated for each gene for each sample [65]. Genes were then clustered based on their FPKM values, which were *Z*-score normalised across all four samples. Heatmap displaying transcription levels for individual genes were produced using pheatmap in the R package v.1.0.12 [66]. Differences in the levels of transcription between samples were established using DESeq2 [67]; genes were considered differentially transcribed if they met the criteria of a false discovery rate (FDR) of <1 × 10^−8^ and a fold change (FC) of ≥2, with ‘upregulation’ defined relative to transcription in samples from female worms.

## 3 Results

### 3.1 Assembly and structure of the genome of *Dirofilaria asiatica*

**Fig. 1:**
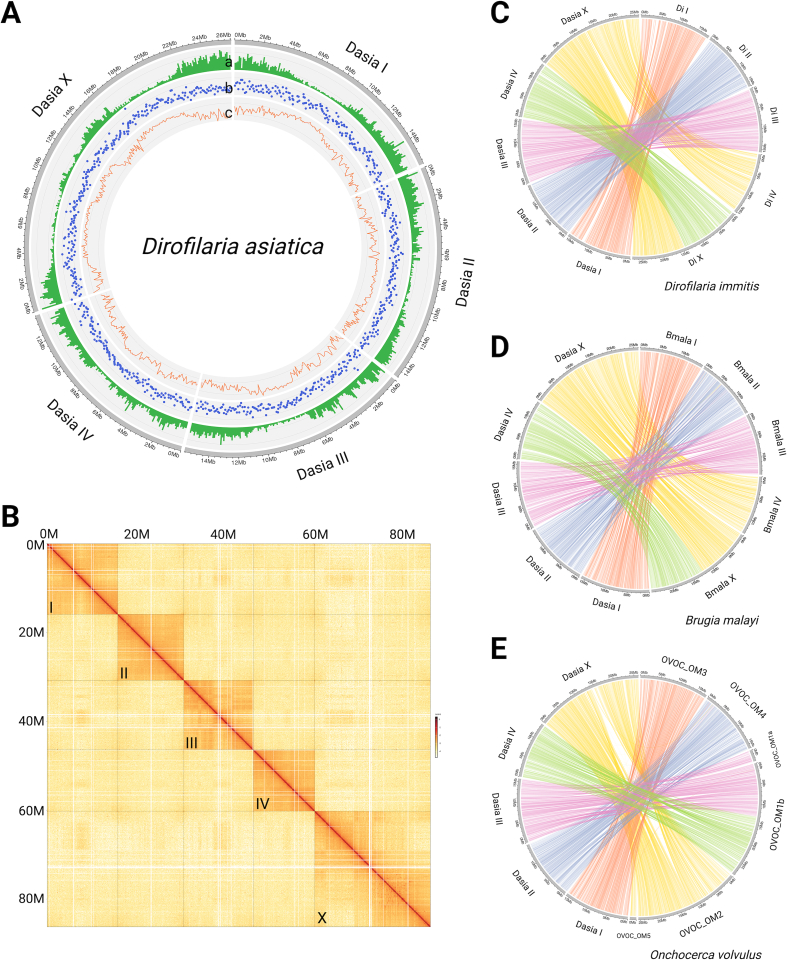
Genome assembly and chromosomal architecture of *Dirofilaria asiatica*, and comparative synteny with *Dirofilaria immitis*, *Brugia malayi* and *Onchocerca volvulus*. (A) Circular representation of the five assembled *D. asiatica* chromosomes, showing distributions of (a) repeat content, (b) gene density, and (c) GC content using a 100 kb sliding window. Chromosomes are colour-coded, with autosomes (Dasia I to IV) and sex chromosome (Dasia X) labelled. (B) Hi-C contact heatmap depicting chromosomal interactions and three-dimensional genome organisation. Strong intra-chromosomal contact domains are visible as dark red diagonal blocks, confirming the integrity of chromosome-scale scaffolding. (C) Syntenic relationships based on 6648 one-to-one orthologues between *D. asiatica* and *D. immitis*. (D) Syntenic relationships based on 6751 one-to-one orthologues between *D. asiatica* and *B. malayi*. Ribbon connections highlight conserved gene order and chromosomal rearrangements across the genomes. (E) Synteny between *D. asiatica* and *O. volvulus* based on 6790 one-to-one orthologues, illustrating conservation and collinearity between the genomes of other filiarial nematodes species studied here (i.e. *D. immitis*, *B. malayi* and *O. volvulus*). (For interpretation of the references to colour in this figure legend, the reader is referred to the web version of this article.)

We assembled a chromosome-level genome of *D. asiatica* using 36 Gb of PacBio long-read sequence data (400-fold coverage) and 133 Gb of Hi-C data (∼1454-fold coverage) obtained from single female and male worms (Table S1). The final assembly spanned 91.9 Mb and comprised four autosomal scaffolds and one sex-linked scaffold, with an N50 of 15.66 Mb, a maximum scaffold length of 26.06 Mb, and a GC content of 27.74 % (Table 1; Fig. 1A, B). To assess genome completeness, we used benchmarking universal single-copy orthologues (BUSCOs) and identified 2966 (94.7 %) of 3131 expected nematode orthologues as complete, with 32 (1.0 %) fragmented and 133 (4.2 %) missing – figures consistent with expectations for parasitic nematodes (Table 1).

**Table 1:** Genome assembly statistics for *Dirofilaria asiatica* and related filarioid nematodes including *Dirofilaria immitis*, *Brugia malayi* and *Onchocerca volvulus*. Key assembly metrics include genome size, chromosome and scaffold counts, N50, GC content, and gaps (Ns). Gene prediction quality was assessed using BUSCO and OMark at the proteomic level.

	*D. asiatica*	*D. immitis*	*B. malayi*	*O. volvulus*
Genome features	(This study)	see [24]	(GCA_000002995.5) d	(GCA_000499405.2)^d^
Genome size (bp)	91,941,431	94,056,743	87,155,713	96,427,137
Number of chromosomes	5	5	5	4
Number of scaffolds	50	79	191	704
Largest chromosome (bp)	26,062,257	27,526,700	24,943,668	28,345,163
N50	15,664,645	15,667,105	14,214,749	25,485,961
GC content	27.74 %	27.78 %	28.42 %	29.19 %
Ns (gaps)^a^	51,400	6800	277,365	3,074,367
Number of gene models	9658	11,852	10,905	12,109
BUSCO – genome (c; s; d; f; m)^b^	94.7; 89.8; 4.9; 1.0; 4.2	97.8; 95.2; 2.6; 0.9; 1.3	99.0; 98.5; 0.6; 0.7; 0.3	98.9; 98.0; 0.9; 0.7; 0.4
BUSCO – proteome (c; s; d; f; m)^b^	91.9; 87.1; 4.8; 1.1; 7.0	93.0; 90.3; 2.7; 0.9;6.1	98.9; 98.3; 0.6; 0.2; 0.9	98.4; 97.3; 1.1; 0.4; 1.3
OMark – proteome (cs; ics; ct; uk)^c^^d^	87.5; 4.1; 0; 8.4	80.7; 5.4; 0; 13.9	87.5; 4.1; 0; 8.4	79.2; 1.7; 0; 19.1

or missing (m) to assess the completeness and quality of a genome assembly;

a Ns is the total number of uncalled bases in the assembly.

b BUSCO results categorise genes into complete (c), complete and single copy (s), duplicated (d), fragmented (f).

c OMark results categorise genes (at the proteome level) into “consistent” (cs), “inconsistent” (ics), “unknown” (uk) or “contaminant” (ct) to estimate the completeness of the gene-repertoire, estimate the proportion of accurate and erroneous gene models or detect possible contamination from other species.

d Reference sequence available via the National Center for Biotechnology Information (https://www.ncbi.nlm.nih.gov/).

We assigned each scaffold a chromosomal identity based on synteny with *B. malayi* (Fig. 1D). Pairwise genome alignments revealed conserved chromosomal architecture between *D. asiatica* and *B. malayi, D. immitis,* and *O. volvulus,* although the degree of structural conservation varied (Fig. 1C-E). The *D. asiatica* genome (91.94 Mb) was slightly larger than that of *B. malayi* (87.16 Mb), but smaller than those of *D. immitis* (94.06 Mb) and *O. volvulus* (96.43 Mb) (Table 1). All three species – *D. asiatica, B. malayi,* and *D. immitis* – possess five chromosomes (four autosomal and one sex-linked), with the X chromosome of *D. asiatica* matching part of X chromosome plus the complete IV autosome of *D. immitis* and *B. malayi* (Fig. 1 C, D). The largest chromosome (X) in *D. asiatica* (26.06 Mb) was marginally smaller than the homologous chromosomes in both *D. immitis* (27.53 Mb) and *O. volvulus* (28.35 Mb), but comparable in size to that of *B. malayi* (24.94 Mb). The GC content in *D. asiatica* (27.74 %) closely matched that of *D. immitis* (27.78 %) and was slightly lower than in *B. malayi* (28.42 %) and *O. volvulus* (29.19 %). The number of predicted protein-coding genes in *D. asiatica* (9658) was also lower than in *B. malayi* (10,905), *D. immitis* (11,852), and *O. volvulus* (12,109) (Table 1; cf. [21,22,24]). These results show that *D. asiatica* has compact and syntenically conserved genome elements and distinctive compositional features relative to the other filarioid nematodes studied here.

### 3.2 The annotated genome

We inferred 9658 protein-coding genes from the *D. asiatica* genome, based on transcriptomic and proteomic evidence (Table 1). We identified initiation and termination codons as well as annotated untranslated regions (UTRs) for 5824 mRNAs. Of the 9658 predicted proteins (Table S2), 8212 (85.0 %) had best matches in the EggNOG database, 6524 (67.6 %) in the GO database, and 7008 (72.6 %) in the KEGG database. Notably, 1446 (14.9 %) proteins showed no detectable homology to any known sequences in these databases. Subsequently, we assessed genome completeness using BUSCOs, identifying 2878 of 3131 (87.1 %) orthologues as complete and 33 (1.1 %) as fragmented (Table 1). The remaining 220 (7.0 %) orthologues were not detected, although whether they are truly absent from this parasitic nematode remains to be verified. We also found that 8.39 % of the genome consists of repetitive elements; these include prominent long terminal repeat (LTR) retroelements [68] and hobo-Activator DNA transposons [69], which we observed predominantly in the central and terminal regions of chromosomes (Fig. 1A).

### 3.3 Parasite-derived molecules (PDMs)

**Fig. 2:**
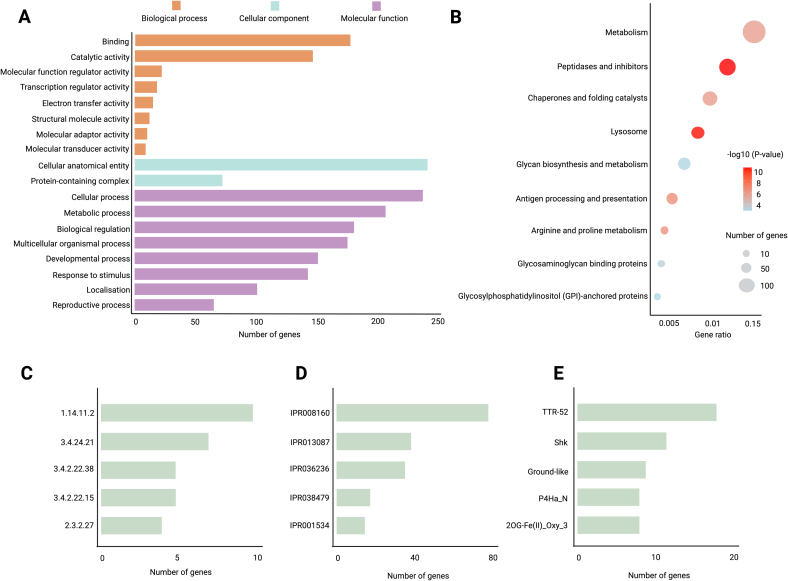
Functional classification of excretory/secretory (ES) proteins inferred from the *Dirofilaria asiatica* genome. Annotation of 881 predicted ES proteins integrates Gene Ontology (GO), KEGG pathways, Enzyme Commission (EC) numbers, Pfam domains, and InterPro entries. (A) GO terms were assigned to 391 proteins (6299 terms), spanning biological process (BP; blue), cellular component (CC; green), and molecular function (MF; red). Top terms included cellular process and metabolic process (BP), binding and catalytic activity (MF), and cellular anatomical entity (CC), reflecting the extracellular nature of many ES proteins. (B) KEGG enrichment (529 proteins, 335 KO terms) revealed significant involvement in pathways including ‘metabolism’ (82 proteins), ‘peptidases and their inhibitors’ (42), ‘chaperones and folding catalysts’ (27), ‘lysosomes’ (25), ‘glycan biosynthesis and metabolism’ (19), and ‘antigen processing and presentation’ (12), among others. Bubble plots indicate gene ratio, gene count and significance. (C) EC numbers were assigned to 145 proteins, dominated by (1.14.11.2; *n* = 10; procollagen-proline 4-dioxygenase; https://enzyme.expasy.org/EC/1.14.11.2). (D) InterPro signatures mirrored Pfam, with top entries including IPR008160 (collagen triple helix repeat), IPR013087 (zinc finger C2H2-type), IPR036236 (zinc finger C2H2 superfamily), IPR038479 (transthyretin-like superfamily) and IPR001534 (transthyretin-like). (E) Most frequent Pfam domains were TTR-52, ShK, Ground-like, 2OG-FeII_Oxy_3 and P4Ha_N, associated with host interaction and proteolysis. (For interpretation of the references to colour in this figure legend, the reader is referred to the web version of this article.)

With a focus on parasite-derived molecules (PDMs) [70] – which are involved in immune evasion and modulation, we predicted a total of 881 excretory/secretory (ES) proteins from the *D. asiatica* genome, representing this species' secretome. Of these, 701 (80 %) were functionally annotated using an integrated workflow (Table S3). GO analysis (level 2) assigned 6299 terms to 391 proteins, spanning 18 biological processes (BPs), two cellular components (CCs) and 14 molecular functions (MFs). The most frequently assigned BPs included cellular processes (GO:0009987; *n* = 238) and metabolic processes (GO:0008152; *n* = 207), while the most common CC and MF terms were cellular anatomical entity (GO:0110165; *n* = 242), binding (GO:0005488; *n* = 178) and catalytic activity (GO:0003824; *n* = 147) (Fig. 2A).

We linked 529 ES proteins (60 %) to 335 KEGG orthology (KO) terms, revealing a significant enrichment in several biological pathways (Fig. 2B; Table S3). Enriched categories included peptidases and inhibitors (*n* = 42, *p* = 6.05 × 10^−11^), lysosomes (*n* = 25, *p* = 7.48 × 10^−11^), antigen processing and presentation (*n* = 12, *p* = 3.45 × 10^−7^), arginine and proline metabolism (*n* = 11, *p* = 4.38 × 10^−7^), chaperones and folding catalysts (*n* = 27, *p* = 1.65 × 10^−6^), and general metabolism (*n* = 82, *p* = 1.91 × 10^−6^). Additional enrichment was detected for glycosaminoglycan binding proteins (*n* = 10, *p* = 5.53 × 10^−4^), glycan biosynthesis and metabolism (*n* = 19, *p* = 9.80 × 10^−4^) and GPI-anchored proteins (*n* = 9, *p* = 1.29 × 10^−3^). The most prevalent Pfam domains among ES proteins were TTR-52 (n = 19), ShK (n = 8), ground-like (n = 11), 2OG-FeII_Oxy_3 (n = 10) and P4Ha_N (n = 10) (Fig. 2E). Comparative analysis showed that 229 (26 %) of the ES proteins were unique to *D. asiatica*, with no detectable homologues in the secretomes of *B. malayi, D. immitis* or *O. volvulus*. Among these, 183 proteins were functionally annotated using EggNOG and InterProScan, while 46 remained uncharacterised (Table S4).

Extending these analyses, we identified a total of six distinct genes encoding CAP domain-containing proteins (i.e. Dasi1G00000001220, Dasi1G00000001951, Dasi1G00000002967, Dasi1G00000007826, Dasi1G00000009015 and Dasi1G00000009363) in the genome of *D. asiatica* (Table S2) – compared with totals of four, eight and 15 such genes in *D. immitis, B. malayi* and *O. volvulus,* respectively [61]. The proteins inferred from these six *D. asiatica* genes matched homologues in *D. immitis, B. malayi, O. volvulus* and *C. elegans,* with the gene pair Dasi1G00000001951 and Dasi1G00000009363 matching the same homologues in each of the other four nematode species studied (Table S2).

### 3.4 Transcriptomic profiling of protein-coding genes, including those encoding the secretome

**Fig. 3:**
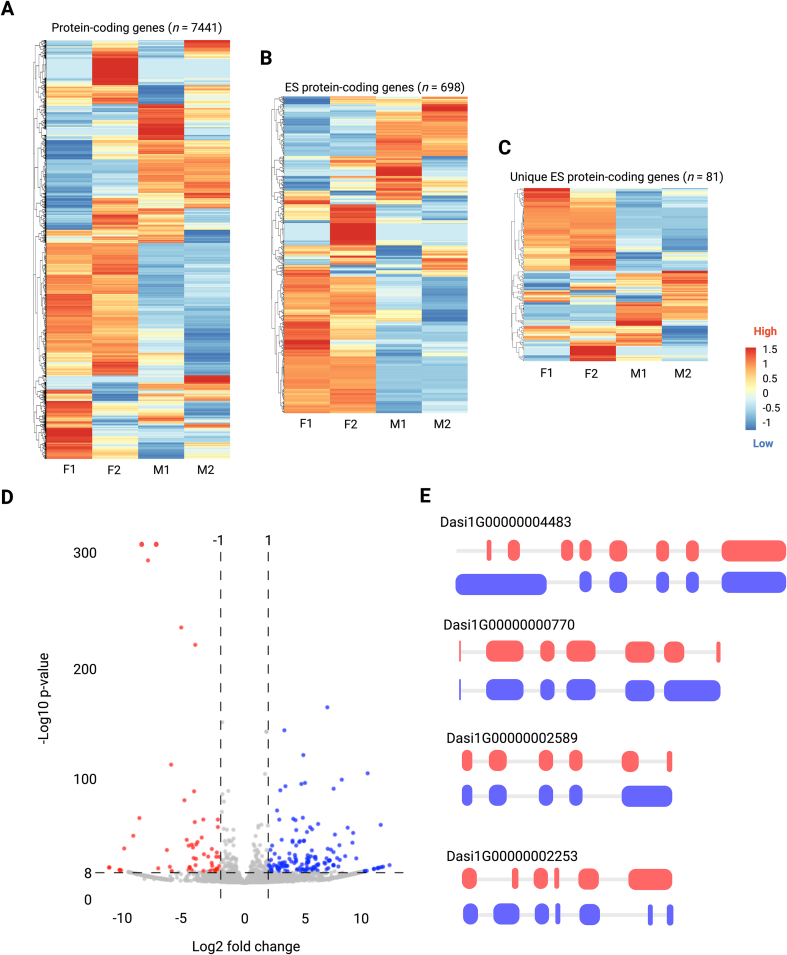
Transcription of protein-coding genes in individual female and male worms of *Dirofilaria asiatica*. (A-C) Transcription profiles of 7411 of the 9658 genes encoded in the genome (panel A); 698 of 881 genes inferred to encode ES proteins (secretome) (panel B); and 81 of 229 genes inferred to encode ES proteins that are unique to *Dirofilaria asiatica* (with reference to *Dirofilaria immitis*, *Brugia malayi*, *Onchocerca volvulus*) in female (F1 and F2) and male (M1 and M2) worms (panel C). The colour scale represents normalised fragments per kilobase of transcript per million mapped reads (FPKM). Genes are clustered based on their transcription profiles (see Materials and methods). (D) A volcano plot showing the differences in transcription between female (red) and male (blue) dots. Differential transcription is indicated by a fold change (FC) of ≥2 and a false discovery rate (FDR) of <10^−8^. (E) Examples of genes exhibiting differential isoform usage in female (red) and male (blue) *D. asiatica*. (For interpretation of the references to colour in this figure legend, the reader is referred to the web version of this article.)

We obtained transcriptomic support for 7411 of all 9658 (77 %) protein-coding genes predicted from the genome (Fig. 3A), including 7050 genes transcribed in females and 6990 genes in males of *D. asiatica* (Table 2; Table S5). Using transcriptomic data for two individual worms per sex, 421 genes were transcribed exclusively in females, and 361 only in males (Table 2).

**Table 2:** Genomic and transcriptomic features of all protein-coding genes – including those encoding excretory-secretory (ES) proteins and ES proteins that are unique to *Dirofilaria asiatica* and not detected in related onchocercids (i.e. *D. immitis, Brugia malayi or Onchocerca volvulus*)*.*

Description Numbers of genes encoding:	Proteins	ES proteins	Unique ES proteins
Total – predicted from the genome	9658	881	229
With current RNA evidence	7411	698	174
With functional annotation	8212	701	183
Transcribed in female and/or male worms	7050 (♀); 6990 (♂)	667 (♀); 716 (♂)	163 (♀); 145 (♂)
Transcribed in either female or male worms	421 (♀); 361 (♂)	81 (♀); 31 (♂)	29 (♀); 11 (♂)
With multiple transcript isoforms	840	75	17
Without canine homologues	5668	450	83

Of all 881 ES protein-coding genes inferred from the genome of *D. asiatica,* 698 (79 %) were transcribed (Fig. 3B), including 667 in females and 716 in males. Of these 881 genes, 81 were transcribed exclusively in females and 31 in males (Table 2; Fig. 3B). Of the 229 genes inferred to encode *D. asiatica*-unique ES proteins, 174 genes were transcribed: 163 in females and 145 in males, with 29 transcribed exclusively in females and 11 only in males (Table 2; Fig. 3C).

Transcript isoform analysis indicated alternative splicing. In total, 840 protein-coding genes were linked to multiple isoforms, including 75 ES protein genes, 17 of which were unique to *D. asiatica.* Sex-associated isoform usage was recorded, with distinct transcript variants observed in one sex but not the other (examples shown in Fig. 3E). The functions inferred for protein genes linked to multiple isoforms are diverse (Fig. 3; Table S6). For instance, gene Dasi1G00000006107 encodes a CP2 transcription factor – implicated in the regulation of gene expression during development and differentiation [71]. Dasi1G00000003247 contains a formin homology 2 (FH2) domain – a structural motif that plays a central role in the reorganisation of the actin cytoskeleton, which mediates essential cellular functions such as motility, adhesion and cytokinesis [72]. Dasi1G00000004804 belongs to the minichromosome maintenance (MCM) protein family, which is critical for initiating DNA replication, contributing to helicase activity and ensuring replication fidelity during the S phase of the cell cycle [73]. In addition, Dasi1G00000004483 encodes proteasome subunit isoforms – part of a multicatalytic proteinase complex that cleaves peptides at specific residues (e.g., Arg, Phe, Tyr, Leu or Glu) under neutral or slightly basic pH conditions – and thus plays an important role in protein turnover and cellular homeostasis [74]. These isoform differences likely contribute to sex-specific physiological and developmental processes or pathways in *D. asiatica.*

## 4 Discussion

This study presents the first chromosome-scale nuclear genome for *D. asiatica* – a recently described species within the genus *Dirofilaria* with zoonotic potential [16]. This high-quality genome enables new insights into the molecular basis of host–parasite interactions, placing *D. asiatica* within a broader genomic and immunobiological framework for filarioid nematodes. Previously characterised only through morphological and mitochondrial sequence data sets [16], *D. asiatica* remained unexplored at the nuclear genomic level. Thus, the genome presented here fills this critical gap and offers a robust resource for a range of fundamental and applied investigations. Comparative analyses with other onchocercids – *D. immitis, B. malayi* and *O. volvulus* – reveal both conserved and lineage-specific features that likely reflect distinct immunomodulatory strategies, developmental biology and host niche adaptation. Through new molecular insights into *D. asiatica*, the genome also contributes to a One Health focus, for which improved understanding of the relationship among the parasite, animals, humans and vectors will be central to monitoring zoonotic risk.

### 4.1 Defining the chromosome-scale genome for *Dirofilaria asiatica*

We successfully generated a highly contiguous nuclear genome for *D. asiatica* from archival ethanol-fixed material using long-read sequencing and Hi-C scaffolding. The final assembly is 91.9 Mb in size and consists of four autosomes and one sex-linked scaffold, reflecting the conserved karyotype of filarioids [75,76]. This chromosome-level resolution enables syntenic comparison with other filarial nematodes and provides a sound structural basis for identifying functional elements and gene family expansions. The genome encodes at least 9658 protein-coding genes, of which the majority is currently supported by transcriptomic data and displays homology with known functional domains. BUSCO and OMark analyses confirmed the completeness and quality of the assembly, and comparative annotation revealed some unique and expanded gene families in *D. asiatica* encoding the secreted and immunomodulatory proteins predicted here. This resource lays the groundwork for future explorations of genetic variation and parasite adaptation at an unprecedented resolution.

### 4.2 Immune modulation and the molecular dialogue with the host animal

Parasitic nematodes, particularly filarioids, survive for extended periods within immunocompetent hosts by deploying highly specialised immune evasion strategies. These strategies include the secretion of a wide array of parasite-derived molecules (PDMs) that modulate innate and adaptive immunity, enabling chronic infection. Geary [70] proposed a conceptual framework in which the molecular “dialogue” between parasite and host is mediated by such PDMs, allowing nematodes such as *D. immitis* to persist in otherwise hostile immune environments. Our study reveals that *D. asiatica* possesses an extensive secretome that is enriched for proteins implicated in immune modulation, many of which relate to known PDMs from other filarial species (Table S2).

Among the parasite-derived molecules (PDMs) likely central to immune evasion in filarial species are cysteine protease inhibitors (cystatins), serine protease inhibitors (serpins), TGF-β homologues and venom allergen-like (VAL) proteins [[77], [78], [79], [80],^86^,^87^]. *D. asiatica* encodes several cystatin-like proteins predicted to interfere with antigen processing and MHC class II presentation (e.g. Dasi1G00000001608, Dasi1G00000001639, Dasi1G00000001746; Table S2), analogous to Bm-CPI-2 in *Brugia malayi,* which inhibits papain-like and asparaginyl endopeptidases to suppress T cell activation [81,82]. The secretome also includes multiple serpin-like protease inhibitors (e.g. SPN-2), encoded by genes such as Dasi1G00000003645, Dasi1G00000008213 and Dasi1G00000008250 (Table S2), homologous to serpins in *B. malayi* that neutralise neutrophil elastase and cathepsin G – key effectors of host inflammatory responses [77,79,83]. In addition, secreted proteins with domains associated with TGF-β signalling and cytokine mimicry (e.g. Dasi1G00000000079, Dasi1G00000001756, Dasi1G00000003027; Table S2) were identified, with homologues in *B. malayi* – such as Bm-TGH-2 and macrophage migration inhibitory factors (Bm-MIFs) – shown to bind host receptors and shift immune responses toward regulatory or alternatively activated states, thereby suppressing pro-inflammatory cytokine production and T cell proliferation [79,80]. The presence of these gene products in *D. asiatica* suggests the use of similar mechanisms to modulate host immunity. In addition, VAL proteins – a subset of CAP domain-containing proteins – are also key molecules in parasitic nematodes and have been implicated in modulating host immune responses, particularly through mechanisms that support immune evasion and parasite survival [77,79,80,86,87]. *D. asiatica* encodes a small number of canonical CAP domain-containing proteins (*n* = 6; Table S2), comparable to *B. malayi* (*n* = 8) and *D. immitis* (*n* = 4), but markedly fewer than *Onchocerca volvulus* (*n* = 15) [61]. This expansion in *O. volvulus* may reflect lineage-specific adaptations linked to distinct host–parasite interactions and unique immune modulatory mechanisms or pathways. Nonetheless, the evolution of CAP domain-containing proteins (both canonical and non-canonical) of filarioid nematodes remains poorly understood, and their specific roles in immune evasion and modulation are yet to be clarified.

Some sex-specific ES proteins might also play a role in immunomodulation in the host animal. Transcriptomic data provided support for 698 ES protein-coding genes, with 81 and 31 transcribed exclusively in females and males, respectively. Of the 229 ES genes identified as unique to *D. asiatica,* 174 were transcribed, including 29 and 11 expressed only in females and males, respectively. These findings suggest potential sex- or stage-associated functional partitioning within the secretome, although interpretation remains preliminary. We also identified 840 genes exhibiting transcript isoform diversity, including 75 ES genes, 17 of which appear to be unique to *D. asiatica.* Several isoform-diverse genes encode known regulatory and structural proteins, such as CP2 transcription factors, MCM family helicases, and proteasome subunits, indicating that transcript-level regulation may contribute to immune modulation and host adaptation. However, transcriptomic analyses conducted here were based on a limited number (n = 4) of worms obtained opportunistically from the definitive host and did not include other developmental stages. Therefore, future research should explore the developmental transcriptome and proteome of *D. asiatica* to elucidate stage-specific gene transcription/expression and clarify the roles of ES proteins – particularly those mediating host immune modulation. Building on these insights, subsequent efforts should focus on annotating PDMs of *D. asiatica* and related filarial nematodes, using advanced structural modelling tools – such as AlphaFold [84,85] – to predict structures and systematically classify, curate and characterise their functions.

### 4.3 Comparative genomics of the secretome: Divergence and conservation

To contextualise these findings, we compared the *D. asiatica* genome with those of other filarioids to assess the evolutionary context of ES gene families and their potential roles in host adaptation. Comparative genomic and synteny analyses indicate that, although *D. asiatica* shares broad chromosomal architecture with *D. immitis* and *B. malayi,* its genome exhibits lineage-specific rearrangements and a distinct suite of ES proteins. The latter repertoire includes 881 predicted ES proteins, of which ∼80 % were functionally annotated. KEGG and GO analyses revealed significant enrichment in immune-relevant pathways including lysosomal function, antigen processing and presentation, glycan biosynthesis and protease inhibition. Notably, *D. asiatica* contains a proportion (26 %) of unique ES proteins that are not shared with *D. immitis, O. volvulus* or *B. malayi,* many of which might represent novel or divergent immunomodulatory factors.

The presence of transthyretin-like (TTR) domains, ShK toxin domains and glycosylated molecules in the *D. asiatica* secretome (e.g., genes Dasi1G00000000220, Dasi1G00000007179 and Dasi1G00000008240; Table S3) suggests that this species has evolved unique mechanisms to interfere with host cell signalling, as also seen in VAL proteins from other filarioids [[77], [78], [79],^86^,^87^]. These distinctions may reflect an adaptation to its primary host niche (subcutaneous tissues) and a broader zoonotic potential. The expansion of such gene families in *D. asiatica,* including ALT-like genes and uncharacterised ES proteins, reinforces the hypothesis that immune evasion has been a significant contributor to genomic innovation in filarial nematodes.

A deeper comparative analysis with *D. immitis, B. malayi* and *O. volvulus* revealed that 229 of the 881 ES proteins (∼26 %) are unique to the secretome of *D. asiatica* (Table 2). Of these 229 unique proteins, 146 share homology with proteins in the dog genome (Table 2), raising the possibility of molecular mimicry or immune tolerance mechanisms that may contribute to the persistent of infection (cf. [[78], [79], [80],^87^]). In contrast, 83 proteins have no known homologues in the dog, indicating that they are parasite-specific and, thus, potential candidates for targeted interventions. The 46 uncharacterised proteins, which lack annotation or known homologues (Table S4), represent a panel of molecules with potentially novel functions. These proteins may include previously unrecognised virulence factors or host-modulatory molecules, and their detailed proteomic characterisation could yield valuable insights into their roles in parasite biology and the specific host–parasite relationship. Taken together, these findings provide evidence that *D. asiatica* possesses a specialised secretome shaped by its unique biological context. A subset of the unique ES proteins in the secretome of this species likely contribute to a complex suite of interactions with its mammalian hosts, combining metabolic adaptation, tissue penetration, immune evasion and transcriptional regulation. Importantly, the subset of unique proteins lacking homology with host proteins or select (and genomically well-characterised) filarial nematodes provides a foundation for research aimed at advancing our understanding of filarial infections and developing species-specific tools for diagnosis and control.

### 4.4 Biological and translational significance

The nuclear genomic resource presented here fills a critical knowledge gap for *D. asiatica* – previously defined using morphological and mitogenomic characters [16]. The capacity to generate a high-resolution genome from archived specimens emphasises the potential for similar studies of other cryptic or neglected filarial taxa (cf. [7]). Our findings provide an essential foundation for functional studies targeting immunomodulatory molecules – some of which may serve as diagnostic markers or immunogenic candidates.

The identification of immunoregulatory proteins bearing functional motifs absent from *C. elegans* but relatively conserved among select filarioid species suggests that some or many of the genes involved have evolved under selective pressures specific to parasitism. The divergent nature of a portion (∼26 %) of *D. asiatica* ES proteins and their proposed roles in immune modulation or evasion indicates the potential for identifying species-specific immunogens that minimise cross-reactivity and enhance protective responses.

In addition to immunobiological investigations, the present genome offers a platform for a range of fundamental and applied future studies. For instance, it can support investigations to define the life history of *D. asiatica* as well as molecular epidemiological research, facilitating the identification of host spectra, transmission patterns and the geographical distribution of *D. asiatica* across Asia. Furthermore, the availability of this genome will enable detailed studies of genetic diversity and structure within and among *D. asiatica* populations across diverse geographic regions and ecological settings. These insights will be critical for the tracking of zoonotic transmission. From an applied perspective, this genome provides a basis for the development of accurate molecular diagnostic tools, capable of distinguishing *D. asiatica* infection from those caused by other filarial nematodes. Such tools are urgently needed to support surveillance and control programs in endemic regions, and to improve differential diagnosis in both veterinary and human medicinal contexts [7,16].

### 4.5 Conclusions

Looking ahead, the genome of *D. asiatica* offers a solid foundation for advancing both fundamental and translational aspects. A key area might be the functional characterisation of predicted ES proteins – particularly cystatins, serpins, and TGF-β-like molecules – to better understand their roles in immune modulation. Stage-specific transcriptomic and proteomic profiling across the life cycle could illuminate developmental processes and host adaptations. This, in turn, might guide experimental studies exploring how *D. asiatica* regulates host dendritic cells, macrophages and T cells during infection. Parallel efforts will be needed to evaluate the immunogenicity potential of conserved and species-specific PDMs in relevant animal (e.g., rodent) models (cf. [70]). Beyond laboratory studies, the genome will support molecular epidemiological and population genomic research to characterise genetic structure, host specificity and transmission dynamics in parts of Asia, where *D. asiatica* is proposed to be widespread. Together, these lines of enquiry should advance our understanding of *D. asiatica* biology, assist in developing novel diagnostic tools and immunogenic candidates as well as provide insights into the evolution of parasitism and adaptive processes in filarial nematodes. Importantly, this genomic resource also provides a basis for a One Health approach to monitoring and controlling dirofilariasis at the human–animal–vector interface.

## CRediT authorship contribution statement

**Neil D. Young:** Writing – review & editing, Writing – original draft, Supervision, Methodology, Investigation, Formal analysis, Conceptualization. **Yuanting Zheng:** Writing – original draft, Visualization, Methodology, Investigation, Formal analysis, Data curation. **Anson V. Koehler:** Writing – review & editing. **Tao Wang:** Writing – review & editing. **Sunita B. Sumanam:** Writing – review & editing, Methodology, Investigation, Formal analysis. **Ushani Atapattu:** Writing – review & editing, Resources. **Bill C.H. Chang:** Writing – review & editing, Methodology, Funding acquisition. **Vito Colella:** Writing – review & editing, Supervision, Resources, Investigation. **Robin B. Gasser:** Writing – review & editing, Writing – original draft, Supervision, Funding acquisition, Formal analysis, Conceptualization.

## Declaration of competing interest

The authors declare that they have no known competing financial interests or personal relationships that could have appeared to influence the work reported in this paper.

## Data Availability

The complete genome assembly is available in the NCBI (National Center for Biotechnology Information) database under accession numbers PRJNA1250938 and SAMN47952083.

## Appendix A Supplementary data

Supplementary Tables S1 to S6 Image 1
